# Micromechanical Punching: A Versatile Method for Non-Spherical Microparticle Fabrication

**DOI:** 10.3390/polym13010083

**Published:** 2020-12-28

**Authors:** Ritika Singh Petersen, Anja Boisen, Stephan Sylvest Keller

**Affiliations:** 1 DNRF and Villum Fonden Center for Intelligent Drug Delivery and Sensing Using Microcontainers and Nanomechanics, IDUN, DTU Health Technology, Technical University of Denmark, 2800 Kgs. Lyngby, Denmark; aboi@dtu.dk (A.B.); suke@dtu.dk (S.S.K.); 2 National Centre of Nano Fabrication and Characterization, DTU Nanolab, Technical University of Denmark, 2800 Kgs. Lyngby, Denmark; 3 Department of Health Technology, DTU Health Tech, Technical University of Denmark, 2800 Kgs. Lyngby, Denmark

**Keywords:** non-spherical microparticle, soft lithography, drug delivery, punching

## Abstract

Microparticles are ubiquitous in applications ranging from electronics and drug delivery to cosmetics and food. Conventionally, non-spherical microparticles in various materials with specific shapes, sizes, and physicochemical properties have been fabricated using cleanroom-free lithography techniques such as soft lithography and its high-resolution version particle replication in non-wetting template (PRINT). These methods process the particle material in its liquid/semi-liquid state by deformable molds, limiting the materials from which the particles and the molds can be fabricated. In this study, the microparticle material is exploited as a sheet placed on a deformable substrate, punched by a robust mold. Drawing inspiration from the macro-manufacturing technique of punching metallic sheets, Micromechanical Punching (MMP) is a high-throughput technique for fabrication of non-spherical microparticles. MMP allows production of microparticles from prepatterned, porous, and fibrous films, constituting thermoplastics and thermosetting polymers. As an illustration of application of MMP in drug delivery, flat, microdisk-shaped Furosemide embedded poly(lactic-co-glycolic acid) microparticles are fabricated and Furosemide release is observed. Thus, it is shown in the paper that Micromechanical punching has potential to make micro/nanofabrication more accessible to the research and industrial communities active in applications that require engineered particles.

## 1. Introduction

In order to influence the biodistribution of drug-loaded micro- and nanoparticles in pharmaceutics and the targeting, release profile, and bioavailability of the drug, the ability to precisely manipulate size, shape, and surface properties of the particles is important [1,2]. Similarly, the composition, size and shape of a metallic nanoparticle dictate the resonant frequency of the electron oscillation in localized surface plasmonics. This phenomenon is used in detecting analytes bound to molecules immobilized on the metallic particle, which is applied in biosensors, immunoassays, and spectroscopy [3,4]. Thus, novel applications in drug delivery, tissue engineering, and biosensing are pushing towards novel fabrication techniques. These techniques can produce specifically designed particles, with additional functionalities and sensitive cargos such as drugs or biomolecules [5,6].

Non-spherical microparticles with precisely controlled physical and chemical properties have been conventionally fabricated by using.

Microelectromechanical systems (MEMS) and semiconductor technologies such as photolithography and dry etching [7,8]. However, these processes are expensive, have low throughput, and can sometimes degrade the cargo in the particle susceptible to UV light or high processing temperatures. Soft lithography is an alternative approach for non-spherical microparticle fabrication [9]. It is an umbrella term for various prototyping methods such as microcontact printing, replica molding, microtransfer molding, micromolding in capillary (MIMIC), solvent-assisted Micromolding, and more recently particle replication in non-wetting template (PRINT) [10,11]. The common feature for all the aforementioned processes is the application of a “soft” deformable mold made of materials such as polydimethylsiloxane (PDMS) for printing, embossing, or molding of the microparticle material. However, such a mold is susceptible to damage already after a few runs of processing. In order to address this limitation for microparticle fabrication, hot punching was developed [12]. In this method, the particle material was prepared on a deformable substrate and patterned by a durable Ni mold instead of a deformable mold [12]. This allowed for large-scale fabrication of high aspect ratio reservoir-based particles for oral drug delivery, declared as microcontainers, in biodegradable and biocompatible polymers [13,14]. However, the application of heat during hot punching of microparticles for drug delivery can lead to degradation of sensitive cargo included in the microparticles such as biomolecule drugs. Furthermore, heating typically implies longer processing time and requires more advanced microfabrication equipment.

Another common feature of soft lithography and hot punching methods is the manipulation of some form of ink or liquid/semi-liquid precursor solution as material for microparticle fabrication. Alternatively, the microparticle material can be a solid sheet that can be deformed and cut to fabricate microparticles with desired structures and dimensions. This new perspective of handling the microparticle layer as a deformable sheet is inspired by a high throughput macromanufacturing technique called punching or blanking that has been used for metal sheet forming for hundreds of years. Blanking and punching are shearing processes in which a punch and die are used to modify webs [15].

Inspired by the punching process, we introduce Micromechanical Punching (MMP) as a simple, benign, and fast process for fabrication of microparticles. Here, we demonstrate the concept and potential of MMP by fabrication of a variety of microparticles with different materials that can potentially be used for a wide array of applications. Finally, as an illustrative example, we show the application of MMP to produce poly(lactic-co-glycolic acid) (PLGA) microparticles loaded with Furosemide (Furo) for oral drug delivery.

## 2. Materials and Methods

### 2.1. Materials

Dichloromethane (DCM) (anhydrous >99.8%), Poly-*ɛ*-caprolactone (PCL) pellets (average M_w_~100,000), Poly(acrylic acid) (PAA) solution (average M_w_~100,000, 35 wt.% in H_2_O), Acetone (>99.5% purity), and gelatin powder were obtained from Sigma-Aldrich (Copenhagen, Denmark). Phosphate buffer saline (PBS) tablets and Furosemide (≥98% purity) acquired from Sigma-Aldrich (St. Louis, MO, USA). Milli-Q water was obtained from MilliQ Integral Water Purication System for Ultrapure Water, manufactured by Merck Millipore (Burlington, MA, USA). Poly(Lactic-co-glycolic acid) (PLGA) 50:50 (acid end cap, M_n_ 85,000–100,000 g/mol) was obtained from Akina, IN, US. Poly(L-lactic acid) (PLLA) 2003D grade is purchased from Natureworks (Minnetonka, MN, USA), with M_w_ = 126,000 Da, determined by size exclusion chromatography measurements. Polytetrafluoroethylene sheets (PTFE sheet of Fluoroplast, opaque) were bought from RS Components A/S. Cellulose weighing paper was acquired from VWR European. Co-extruded polyethylene (PE) and polypropylene (PP) films were obtained by Inmold A/S.

### 2.2. Scanning Electron Microscopy and Stylus Profilometry

A TM3030Plus (Hitachi, Japan) tabletop SEM was used for scanning electron microscopy (SEM). The micrographs were obtained at an operating voltage of 15 KV in charge reduction mode using mixed secondary electrons (SE) and backscattered electron (BSE) detector signal.

The thicknesses of the PLGA-Furo films were measured using KLA-Tencor Alpha Step IQ stylus profilometer (Milpitas, CA, USA). The thickness of the film was measured by mechanically removing a part of the coated area to expose the substrate beneath. Then the thickness was measured by doing step height analysis. An average of five thickness measurements on the PLGA-Furo film is reported here.

### 2.3. Film Production Using Blade Coating

For blade coating, first, a homogenous solution of polymer or polymer–drug was prepared. The homogenous solution of biocompatible and biodegradable polymers like PCL, PLLA, and PLGA was prepared as 10 wt.% in DCM solvent. The water soluble polymers like gelatin and PAA were dissolved as 5 wt.% concentration solutions. These solutions were dispensed on an Erichson blade coating machine, and the blade height was adjusted as per the thickness required. PCL, PLLA, and PLGA solutions were coated with blade height ≈ 0.5 mm, while gelatin and PAA solutions were coated with blade height ≈ 0.1 mm. The blade was then moved at a slow speed of 0.5 m/min. The film was left overnight for solvent evaporation. The films were either directly prepared on the deformable substrate or were coated on a PTFE sheet and peeled later.

In order to prepare PLGA-Furo films for the in vitro release of Furosemide from PLGA, a homogenous solution of 10 wt.%, consisting of 1:5 Furosemide to PLGA in acetone and DCM, was prepared. The solution was blade coated on a PTFE sheet and left overnight at room temperature for solvent evaporation. This resulted in a 40 ± 4 µm thick PLGA-Furo film. The film was peeled from the PTFE sheet and transferred to a PP sheet.

### 2.4. Preparation of Formulation: Capsule Loading

First the lid of the rat capsule is weighed without any microparticles. Then, the cap of the capsule is taken off and the capless capsule is placed in a capsule holder. The particles lying on the deformable substrate were scraped off by a lab knife and gently nudged in a funnel that sits on the top of a stainless steel holder. The holder is specifically designed for the capsule in terms of shape and size. A funnel is then placed on this holder and then the microparticles are scraped off the deformable substrate with the help of a lab knife. The scraped microparticles are allowed to fall on the funnel in order to fill the capsule. Sometimes the microparticles clogging the funnel hole are gently nudged in the capsule by a piston. During and after the filling of the capsule, it is weighed with the cap on. Approximately 0.5 mg of particles was weighed in the capsules for the in vitro release studies.

### 2.5. In Vitro Release of Furosemide from PLGA Matrix

In vitro drug release was measured in dissolution studies performed with a microdissolution profiler [16]. The PLGA-Furo particles were filled in the rat gelatin capsules and placed in the glass vials filled with 10 mL of phosphate buffer (pH 7.5) solution (PBS). The gelatin capsules are predominantly dissolved within few seconds of being placed in the PBS media. Experiments were performed at 37 °C using a stirring rate of 200 rpm. The path length of UV probes was chosen as 20 mm on the µDiss profiler (Pion, United Kingdom). Standard curve for the calibration of the channels was performed prior to the release experiments. For the standard curve, Furosemide in milliQ (pH = 10) stock solution was prepared. A fixed amount of this solution was added to 10 mL of PBS. The defined concentration was measured by detecting the UV absorbance from the added analyte in the stock solution. After the calibration of the channels, the actual experiment was performed with the capsules by scanning the samples by the in situ UV probes every 30 s with a run time of 7 h. The release experiments are expressed as normalized (to the maximum drug released after 7 h) drug release over time. The experiments were performed in 6 replicates from 3 independent productions within a week of film and particle formation.

### 2.6. Micromechanical Punching (MMP)

**Figure 1:**
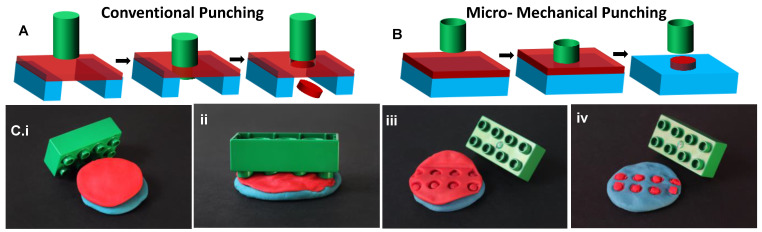
(**A**) Conventional punching vs. (**B**) micromechanical punching; (**C**) do-it-yourself punching using Playdough^®^ and Lego^®^ block showing the basic steps of the MMP process: (i) sample preparation, (ii) MMP, (iii) demolding the mold from the microparticle layer, and (iv) microparticles obtained on the deformable substrate after peeling off the surrounding punched film (Green: Hard mold; red: Microparticle layer; blue: Deformable substrate).

The conventional punching or blanking process includes three basic steps illustrated in Figure 1A: (1) A metal sheet is positioned on an inflexible die. (2) The punch and the metal sheet are brought into contact by the application of a high force. The penetration of the punch into the voids of the die results in build-up of stresses in the metal sheet with the maximum stress located at the edges of the punch. When the maximum stress exceeds the ultimate tensile strength of the metal, the metal sheet below the punch is cut free from the surrounding metal. (3) Finally, the punch and the die are separated [15].

The two significant differences of the proposed MMP process compared to the conventional punching technique are the utilization of a deformable substrate and a hollow punch. In MMP, the microparticle layer is placed on a deformable substrate in the form of a solid material sheet. This flexible stack of the substrate and the microparticle layer is punched by a hard but hollow punch or mold. During MMP, the cutting or indentation of the microparticle layer by the hollow mold and the final punching of the microparticle layer occur in a single step, leading to the fabrication of microparticles of controlled shapes, sizes, and surface topography (Figure 1B,C).

The MMP process starts with the preparation of the deformable substrate, the microparticle layer, and the hard mold (Figure 1(Ci)). For the deformable substrate, compression molded or extruded polymer sheets, solution-casted cross-linked PDMS layers, commercially available aluminum (Al) sheets, or polymer films prepared from solutions by spin coating, blade coating, or spray coating are suitable [17]. As high pressure is applied during mechanical punching, a robust deformable substrate is important. For example, a PDMS substrate succumbs under 5 bars platen pressure while a polyethylene (PE) substrate can tolerate platen pressures >20 bars. The microparticle layer can be materials, ranging from polymers and gels to metals. At room temperature any material which is not too ductile as poly-ε-caprolactone and too brittle as ceramics can be mechanically punched. However, in principle, ductile materials can be frozen and brittle materials can be processed at elevated temperatures for successful punching. The microparticle layer can be directly deposited on the deformable substrate by above mentioned coating techniques or prepared separately and then transferred to the deformable substrate before MMP. Finally, MMP requires a robust mold that does not succumb under the pressure applied. The mold consists of large arrays of protruding walls around a cavity of desired size and shape, as shown in Figure 1(B,Ci). In general, the mold material should have a higher hardness than the other materials in the stack. Molds with sharp protrusions are required for easy punching, while mold features with low roughness and slightly positive sidewall angles are necessary for easy demolding after the MMP process. The mold protrusions should be higher than the thickness of the material layer to allow for complete punching.

After preparation of the mold, the material layer and the deformable substrate are prepared. The complete stack is placed in a parallel plate (P2P) embosser. High pressure is applied on the stack in order to punch the microparticle layer (Figure 1(Cii)). After the punching process, the mold is separated from the microparticle layer (Figure 1(Ciii)). The leftover film in between the punched particles is peeled from the substrate (Figure 1(Ciii)) and the microparticles either remain on the deformable substrate (Figure 1(Civ,D)) or are transferred to the mold. In the latter case, another step of bonding or particle transfer to a desired substrate is performed. The micromechanical punching was performed using the hot embosser (Collin^®^ Press, Ebersberg, Germany, 300 SV). The particles were generally punched at pressures >20 bars for 1 min.

## 3. Results and Discussion

### 3.1. Fabrication of Microparticles with Various Materials, Shapes and Sizes

**Figure 2:**
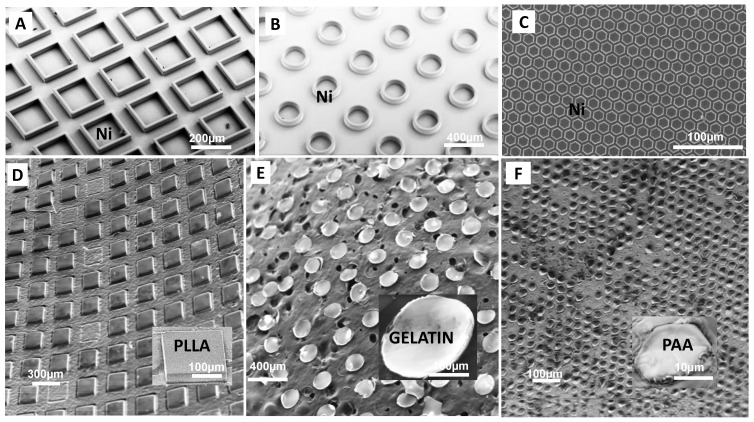
(**A**–**C**) SEM micrographs showing the Ni molds used for MMP; (**D**) square microparticles punched from poly-l-lactic acid polymer film; (**E**) circular microparticles punched from gelatin film; and (**F**) hexagonal particles in water soluble polymer PAA with order magnitude smaller sizes.

For the demonstration of the MMP process, a Ni mold with protruding walls, as shown in Figure 2A, was fabricated by using a dry etching, electroplating, and molding (DEEMO) process [18]. A molecular layer of perfluorodecyltrichlorosilane (FDTS) was deposited on the Ni mold using molecular vapor deposition (MVD). This improves the separation of the mold and the punched polymer microparticles by reducing the adhesion forces between the two materials. MMP was performed at platen pressures ≥20 bars for 1 min. Figure 2A–C shows Ni molds used in MMP with protrusions of square, circular, and hexagonal shapes, respectively. The diagonal of the square microstructures on the mold has a length 200 µm. The height of the protrusions is 70 µm and the thickness of the walls is 20 µm. The diameter of the circular microstructures on the mold is 260 µm. The height of the protrusions is same as the square microstructures, that is, 70 µm while the wall thickness is 40 µm. The hexagonal Ni stamp shown in the Figure 2C has protrusions of height = 20 µm, diagonal length = 20 µm, and wall thickness = 4 µm.

As an initial proof of concept, MMP was performed on uniform single material microparticle layers such as metal sheets and polymer films. These films had thicknesses measured in the range of 10 µm to 60 µm. To evaluate punching of biodegradable and biocompatible polymers, 14.7 wt.% of Poly-l-lactic acid (PLLA) solution in dichloromethane (DCM) was blade-coated on a polytetrafluoroethylene (PTFE) sheet, peeled off the PTFE surface and then placed on a PE substrate layer for punching. MMP was performed and square shaped PLLA particles were obtained on the deformable PE layer as shown in Figure 2D. Figure 2E shows a punched and dried gelatin layer lying on a carbon tape. The gelatin layer was obtained by blade coating of a 20 wt.% aqueous collagen solution on an oxygen plasma treated PP sheet and drying the film overnight. The gelatin layer was punched, after which the gelatin particles remained attached to the Ni mold and were finally harvested on a carbon tape. In order to fabricate smaller microstructures, an aqueous solution of 15 wt.% Poly Acrylic Acid (PAA) was coated on a plasma treated PP sheet using blade coating. The wet film was left overnight, which was later on punched by Ni mold having hexagonal protrusions of height = 20 µm, diagonal length = 20 µm, and wall thickness = 4 µm. The punching was performed under a higher platen pressure of 40 bars for 1 min to accommodate for the smaller sizes. PAA hexagonal microparticles were obtained on the PE film after demolding as shown in Figure 2F.

### 3.2. Fabrication of Microparticles from Pre-Textured Films: Naturally Occurring or Artificially Patterned

**Figure 3:**
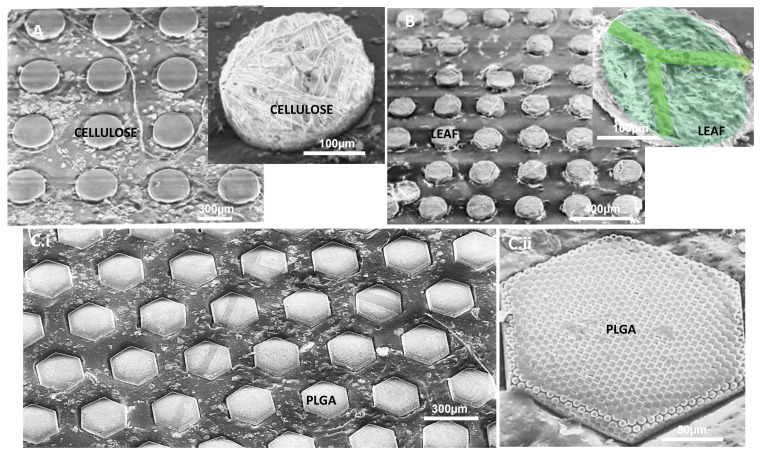
Mechanical punching: (**A**) microparticles from cellulose paper showing the fibrous structure of the microparticle, (**B**) microparticles from dried fall leaf showing the leaf patterns, and (**C**) hexagonal hierarchal microparticles.

Fabrication of microparticles from polymer films with nanostructures, surface texture, or those consisting of porous materials with processes such as soft lithography or PRINT is challenging. MMP is ideal for producing microparticles from such pre-patterned films. In Figure 3A, microparticles punched out of cellulose paper on extruded polypropylene (PP) film as deformable substrate are shown. The close-up of the particles shows that the fibrous structure of the paper was maintained after the punching process. To demonstrate that this process can be further applied to fabricate particles from natural materials displaying patterns, a dried beech hedge leaf (*Fagus sylvatica*) was punched while lying on a PP sheet. Figure 3B depicts the retention of the leaf patterns on the fabricated particles. Figure 3(Ci,Cii) shows hexagonal-in-hexagonal hierarchical particles. These particles were fabricated from a poly(lactic-co-glycolic acid) (PLGA) film which was obtained by blade coating 10 wt.% PLGA solution in DCM on a PTFE sheet. The coated solution was dried overnight. The resulting film was first hot embossed at 80 °C for 5 min by a Ni mold with honeycomb hexagonal structures with a feature size of 2 µm. Afterward, the patterned film was removed from the PTFE sheet and placed on a PE film. This stack of PLGA and PE films was mechanically punched by the Ni mold with hexagonal features. These hexagons have diagonals of 250 µm in length, height of walls = 70 µm and wall thickness = 20 µm. Punching of the prepatterned film with this mold results in particles with hierarchical structures.

### 3.3. Micromechanical Punching of Drug-Loaded Microparticles

One of the major advantages of MMP lies in the production of regular and uniform particles as compared to the conventional methods for the production of spherical particles which usually have a broad particle size distribution. Moreover, MMP leads to the fabrication of flat asymmetric particles that can resist the peristaltic flow of intestinal fluids and allow better contact between the mucosa walls. This leads to better adhesion and retention of the asymmetric particles and hence, higher bioavailability of the drug [19]. Here, flat PLGA-Furo microparticles are fabricated using MMP. Furosemide is a loop diuretic usually found in a stable crystalline form with very low solubility in aqueous media at room temperature. It has been previously proven that drugs dispersed in solid polymer matrices can stay in stable amorphous forms, increasing the bioavailability of the drug [20]. Therefore, Furosemide is embedded in PLGA polymer matrix as microdisk-shaped PLGA-Furo microparticles.

**Figure 4:**
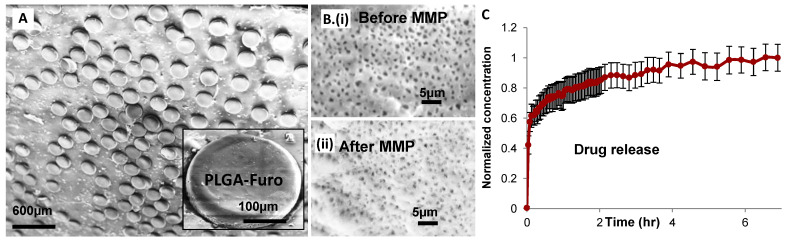
PLGA-Furo microparticle fabrication and characterization: (**A**) PLGA-Furo microparticles lying on the PP substrate after the MMP process; (**B**) porosity of the blade coated PLGA-Furo films before (i) and after MMP (ii) showing that the film remains porous after the punching process; and (**C**) in vitro release of Furosemide from the PLGA microparticles.

For the fabrication of PCL-Furo flat microparticles, the PLGA-Furo film was punched with 20 bars for 1 min. Figure 2C depicts the Ni mold and Figure 4A illustrates the punched PLGA-Furo particles on the PP substrate. Figure 4B shows that the punched microparticles remain porous after the MMP process.

After the drug-loaded microparticles were fabricated, they were filled in gelatin capsules for release studies. Figure 4C shows a dissolution profile where the drug concentrations are normalized with respect to the maximum drug concentration after 7 h of dissolution study. The drug release occurs primarily through diffusion as 5–7 h is not long enough to lead to the degradation of PLGA in PBS buffer (pH = 7.5) [14]. The drug release profile in Figure 4C represents an initial burst release where approximately 50% of drug is release within first 5 min. The high burst release can be attributed to a Furosemide rich surface and the amorphous form of Furosemide in the PLGA-Furo microparticles. During the PLGA-Furo film formation, the solvent evaporation of film lead to Furosemide getting distributed closely to the surface of the film [21]. As there is no further movement of Furosemide in the solidified film during the MMP process, Furosemide-rich surface in the microparticles is obtained. Moreover, Furosemide is quenched in its amorphous form due to the rapid evaporation of low boiling point solvent, Dichloromethane [21]. With no heat applied during the MMP process, Furosemide dispersed in the matrix of PLGA microparticles continues to stay in its amorphous form. After the initial burst release, the remaining drug release from the deeper layers of the polymer matrix becomes slow. These experiments demonstrate that the microparticles fabricated by MMP can be utilized in multiparticulate drug delivery systems [22].

## 4. Conclusions

In this paper, the Micromechanical Punching (MMP) process is introduced for the fabrication of microparticles. Microparticles of various shapes and sizes, made up of biodegradable and biocompatible polymers like PCL, PLGA, and PLLA and water-soluble polymers like gelatin and PAA, have been successfully fabricated using MMP. It is also demonstrated that MMP can be used for punching prepatterned, fibrous or porous films to successfully fabricate hierarchal/patterned, fibrous, or porous microparticles, respectively. Some of the examples shown in the paper are punching of fibrous cellulose sheet, *Fagus sylvatica* leaf and hexagon- patterned PLGA films. Finally, using MMP, Furosemide (Furo)-containing PLGA microparticles are fabricated with uniform size and shape. In vitro release studies are conducted on these PLGA-Furo particles. Successful release of Furosemide from PLGA matrix is shown depicting the viability of using MMP for drug delivery applications.

MMP is a low cost, single-step process that can be scaled up to roll-to-roll production, which in principle can be completely solvent-free. In future, it would be interesting to develop this technology to fabricate nanoparticles that can be applied for future applications in biotechnology and photonics. Investigation into the effect of pressure on thin film materials, 2D materials, and sensitive cargos embedded in the films will be interesting as well as required for applications like drug delivery and tissue engineering. Micromechanical Punching has potential to obtain widespread application in academic laboratories and industrial scale manufacturing around the world due to the simplicity of its execution on practically any conceivable material that can be acquired as a sheet or can be applied on a substrate film.

## Data Availability

Data is contained within the article.
